# Identifying ENO1 as a protein target of chlorogenic acid to inhibit cellular senescence and prevent skin photoaging in mice

**DOI:** 10.1111/acel.14433

**Published:** 2024-12-31

**Authors:** Xueling He, Chen Wang, Qianyu Zhang, Tong Yang, Qiuyan Guo, Yaxu Wang, Jiayue Guo, Pengjie Wang, Junzhe Zhang, Huan Tang, Yinhua Zhu, Jigang Wang

**Affiliations:** ^1^ State Key Laboratory for Quality Ensurance and Sustainable Use of Dao‐di Herbs, Artemisinin Research Center, and Institute of Chinese Materia Medica China Academy of Chinese Medical Sciences Beijing China; ^2^ Department of Urology, The Second Clinical Medical College Jinan University (Shenzhen People's Hospital) Shenzhen Guangdong China; ^3^ Beijing Advanced Innovation Center for Food Nutrition and Human Health, Department of Nutrition and Health China Agricultural University Beijing China; ^4^ State Key Laboratory of Antiviral Drugs, School of Pharmacy Henan University Kaifeng China

**Keywords:** activity‐based protein profiling, chlorogenic acid, enolase 1, glycolysis, photoaging

## Abstract

Cellular senescence plays a critical role in repeated ultraviolet (UV) exposure‐induced skin photoaging. Currently, from the perspective of regulating senescent cells, potent compounds or reliable protein targets that could effectively prevent skin photoaging have not yet been reported. Herein, we demonstrated that chlorogenic acid (CGA) significantly inhibited UVA‐induced senescence of human dermis skin fibroblasts (HDF) cells by screening the natural product library. The activity‐based protein profiling (ABPP) result revealed that Enolase 1 (ENO1) is one of the direct targets of CGA in HDF cells. Further mechanism research indicated that CGA covalently binds to ENO1, and prevented UVA‐induced cellular senescence by suppressing the activity of ENO1 protein to block the glycolytic pathway. Importantly, we found that CGA dose‐dependently reduced the skin wrinkle score, alleviated skin pathological features and inhibited senescent characteristics in a photoaging mouse model. The proteomic analysis revealed that CGA treatment effectively inhibited senescence‐associated secretory phenotype (SASP) secretion and glycolysis in skin samples of mice. Collectively, our study not only demonstrated that inhibiting cell senescence is an effective anti‐skin photoaging strategy, but also revealed that ENO1 is a promising protein target to prevent photoaging.

## Graphical abstract

A schematic depiction of chlorogenic acid inhibited UVA‐induced cellular senescence and mouse skin photoaging by suppressing the activity of Enolase 1 (ENO1) protein to block glycolytic pathway.

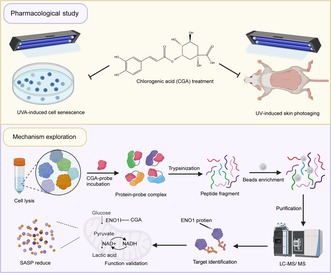

**Abbreviations**
ABPP: activity‐based protein profiling
CETSA: cell heat transfer analysis
CGA: chlorogenic acid
CGA‐P: CGA chemical probe
ECAR: extracellular acidification rate
ECM: extracellular matrix
ELISA: enzyme‐linked immunosorbent assay
ENO1: Enolase 1
GR: granulocyte
H&E: hematoxylin and eosin
HDF: human dermis skin fibroblasts
IAA: iodoacetamide
IAA‐P: IAA probe
IL‐6: interleukin‐6
LD: lactate dehydrogenase
Lym: lymphocyte
MMPs: matrix metalloproteinases
PA: pyruvic acid
PDK4: dehydrogenase kinase
RA: rapamycin
ROS: reactive oxygen specie
RT‐PCR: real‐time fluorescent quantitative PCR
SASP: senescence‐associated secretory phenotype
siRNA: small interfering RNA
TIMPs: tissue inhibitors of matrix metalloproteinases
TMT: Tandem Mass Tag
TNF‐α: tumor necrosis factor α
UV: ultraviolet
WBC: white blood cell

## 1 INTRODUCTION

Photoaging is a skin aging process caused by long‐term exposure of the skin to ultraviolet (UV) radiation and is closely related to the occurrence of skin cancer (Bellei & Picardo, 2020). Applying sunscreen is one of the most important means to prevent photoaging by blocking the damage of ultraviolet rays (Guan et al., 2021). The main methods to prevent or treat skin photoaging include drug therapy (including vitamin C, vitamin E, coenzyme Q10, retinoids and so on), chemical exfoliation, microwave therapy and laser therapy, and so forth. (Rabe et al., 2006). However, these existing therapies only alleviated skin photoaging to a certain extent, and all have certain defects. Therefore, it remains a critical need to fully understand the mechanism of photoaging and develop drugs or therapeutic strategy to treat or prevent skin photoaging.

The skin is organized into three layers: the epidermis, the dermis, and the subcutaneous tissue (Wong et al., 2015). Long‐wave ultraviolet UVA (320–400 nm) could penetrate the epidermis into the dermis and significantly affect human dermal fibroblasts (HDF), the main cell type of the dermis structure (Lan et al., 2019). Therefore, HDF has attracted a lot of research attention when exploring the mechanism of skin photoaging. Currently, the specific molecular mechanism underlying photoaging involves UVA‐induced elevation of reactive oxygen species (ROS) level, which disrupts the TGF‐β/Smad signaling pathway, leading to reduced collagen synthesis in HDF (Liu et al., 2019). Additionally, the dysregulated expression of matrix metalloproteinases (MMPs) and tissue inhibitors of matrix metalloproteinases (TIMPs) in HDF results in increased extracellular matrix (ECM) degradation (Shin et al., 2019).

In recent years, the important roles of senescent cells during skin photoaging process have been revealed, providing a novel insight to understand photoaging (Salminen et al., 2022). Senescent cells are characterized by dysfunctionality, an arrested cell cycle, and the secretion of a plethora of factors that are generically referred to as senescence‐associated secretory phenotypes (SASPs) (Cai et al., 2022; Coppé et al., 2010; López‐Otín et al., 2013). SASPs include cytokines, chemokines, and various proteases, which create a chronic inflammatory microenvironment, speed up the aging process, and lead to age‐related diseases (Childs et al., 2017). Both senescence and SASP are sensitive to cellular and organismal metabolic states, which can drive phenotypes associated with metabolic dysfunction (Wiley & Campisi, 2021). During photoaging, UV radiation can trigger cellular oxidative stress and inflammation, and may also affect the metabolic activity of cells (Hill & Van Remmen, 2014). A previous study has indicated that UV radiation may affect the glycolytic pathway of skin cells, resulting in the accumulation of glycolytic products and intracellular metabolic disorders, thus accelerating the occurrence of skin aging (Snaidr et al., 2019).

Chlorogenic acid (CGA), an important bioactive dietary polyphenol, is found in high content in various polyherbs and foodstuffs, such as eucommia, honeysuckle, green coffee beans, potatoes, apples, and tea leaves. It has many pharmacological effects, including anti‐oxidation and immune regulation by reducing ROS production and inflammatory cytokine release (Naveed et al., 2018). Recent studies have shown that CGA can regulate collagen metabolism in UVA‐irradiated human dermal fibroblasts (Xue et al., 2022), suggesting its potential application in the treatment of skin photoaging. In addition, CGA has also been reported to regulate glucose, lipid metabolism and glycolysis (Meng et al., 2013; Wang et al., 2023). However, specific targets and molecular mechanisms by which CGA prevents photoaging by regulating glycolysis have not been clarified.

In this study, we have screened CGA from a natural product library in our laboratory and demonstrated that CGA significantly inhibited UVA‐induced senescence of HDF cell. The activity‐based protein profiling (ABPP) technology revealed that Enolase 1 (ENO1) is the direct target of CGA in HDF cells. Further mechanism research indicated that CGA inhibited UVA‐induced cellular senescence by suppressing the activity of ENO1 protein to block the glycolytic pathway. Importantly, we found that CGA dose‐dependently reduced the skin wrinkle score, alleviated skin pathological features, and inhibited senescent characteristics in a photoaging mouse model. The proteomic analysis revealed that CGA treatment effectively inhibited SASP secretion and glycolysis in skin samples of mice.

## 2 RESULTS

### 2.1 Screening of active molecules to prevent UVA‐induced senescence of HDF cells

**FIGURE 1:**
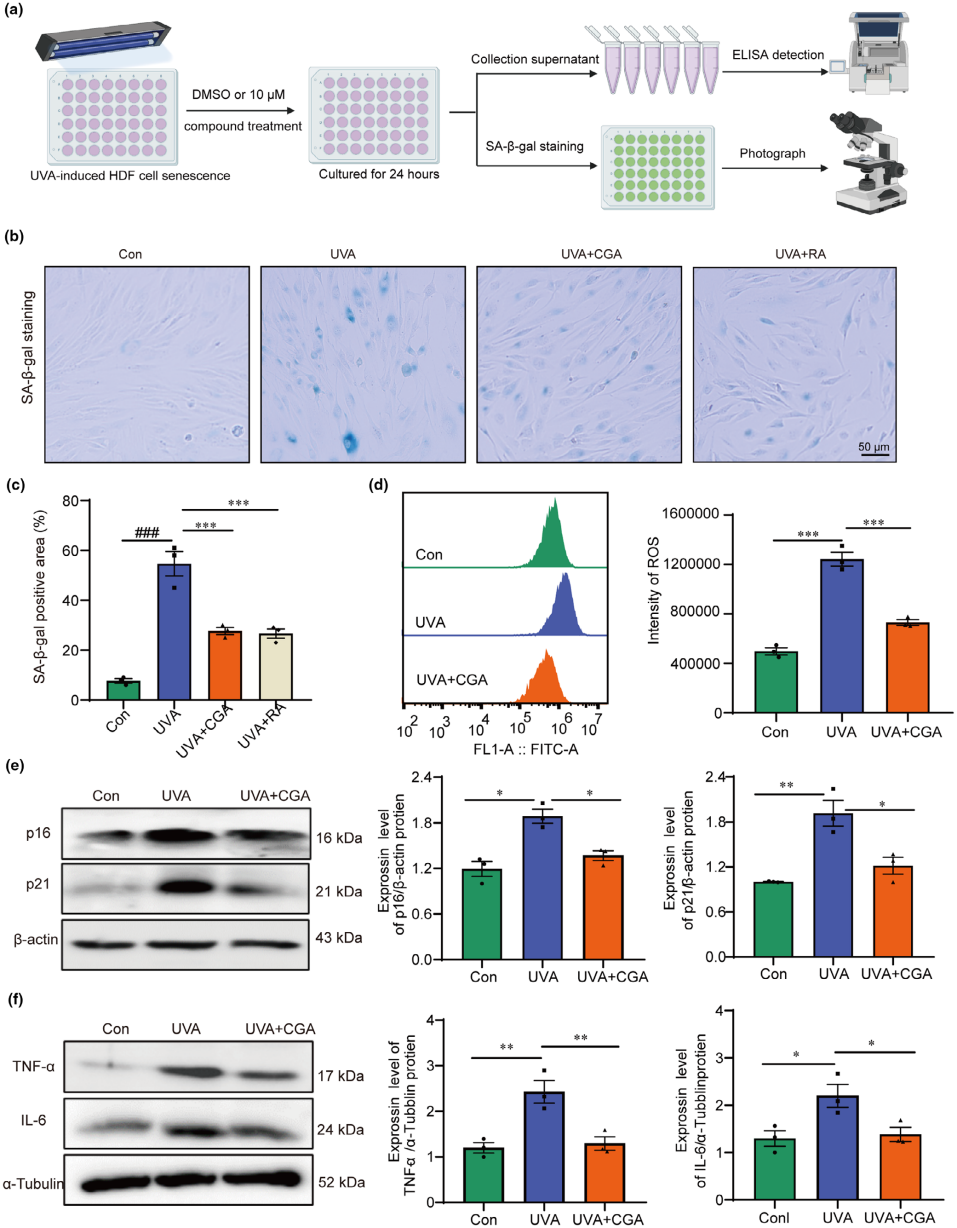
Chlorogenic acid prevents UVA‐induced HDF cells senescence. (a) A flowchart shows potential drugs for screening against photoaging. (b, c) The representative graphs of SA‐β gal staining and statistical result of SA‐β gal positive cell number in Con, UVA, UVA + CGA and UVA + RA group (scale bar = 50 μm, *n* = 3 samples per group), ***p < 0.001. (d) Flow cytometry shows the ROS level and statistics in Con, UVA, UVA + CGA group, (*n* = 3 samples per group), ***p < 0.001. (e) The expression of p16 and p21 protein and statistical result in Con, UVA and CGA group (*n* = 3 samples per group), *p < 0.05, ***p < 0.001. (f) The expression of TNF‐α and IL‐6 protein and statistical result in Con, UVA and CGA group (*n* = 3 samples per group), *p < 0.05, **p < 0.01.

Candidate compounds against UVA‐induced photoaging were screened from 27 natural products at a concentration of 10 μM. The immortalized HDF cells were treated with UVA irradiation for 30 min to induce senescence, and 10 μM natural products were added to the medium and incubated with the cells for 24 h, as Figure 1a depicted. Senescent cells were characterized by elevated SA‐β‐gal activity, SASP secretion and increased p16 and p21 protein expression (Jin et al., 2024; Lee et al., 2006; Mohamad Kamal et al., 2020). We then evaluated the effects of these compounds on cellular senescence by performing SA‐β‐gal staining and measuring the content of the representative SASP factors (TNF‐α and IL‐6) in the cell supernatant by ELISA. The results indicated that CGA could effectively reduce the proportion of SA‐β‐gal positive cells induced by UVA treatment (Figure S1a,b). The ELISA data confirmed that CGA exhibited the best biologic activity in inhibiting the senescence of HDF, supported by the decreased TNF‐α and IL‐6 secretion in treated groups (Figure S1c,d).

Furthermore, CGA is just as effective as rapamycin (RA, a positive drug that suppress senescence, concentration in 5 nM) in preventing UVA‐induced senescence of HDF cells (Figure 1b,c) (Olga & Leontieva, 2017). Flow cytometry results showed that CGA also inhibited UVA‐induced elevation of ROS level (Figure 1d). And Western blot analysis further showed that CGA could effectively inhibit the expression levels of p16 and p21 proteins in UVA‐induced HDF cells (Figure 1e). Moreover, CGA can also significantly suppressed the expression levels of inflammatory factors TNF‐α and IL‐6 proteins in UVA‐treated HDF cells, in line with the above ELISA data (Figure 1f). In conclusion, these results demonstrated that CGA effectively prevented UVA induced senescence of HDF in vitro.

### 2.2 Identify the protein targets of CGA in senescent HDF by ABPP technology

**FIGURE 2:**
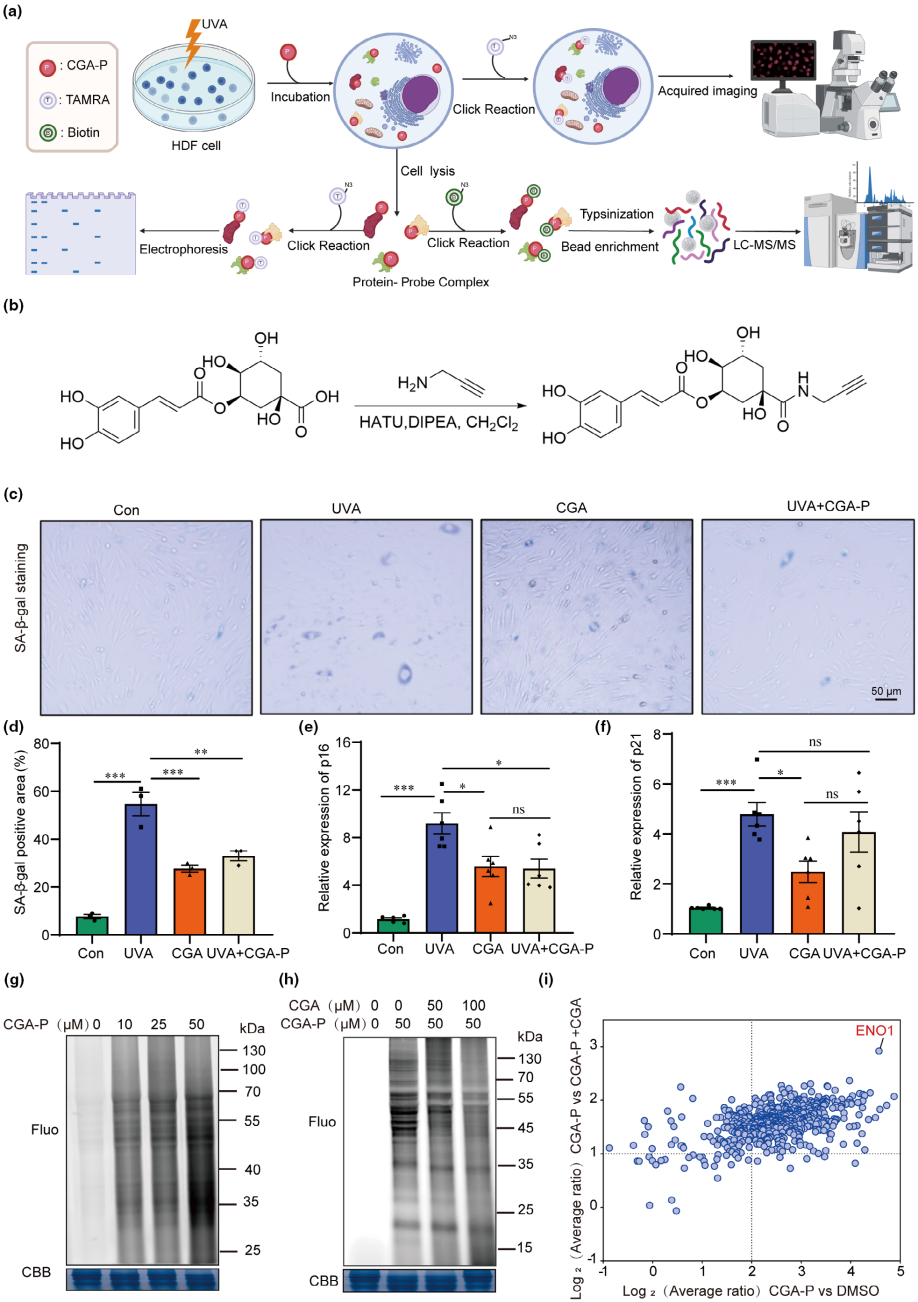
ABPP combined with LC–MS/MS methods to analyze and identify targets for chlorogenic acid inhibition of UVA‐induced photoaging. (a) A flowchart shows the process of identify targets by ABPP. (b) Synthetic process and chemical structure of CGA‐probe. (c, d) The representative graphs of SA‐β gal staining and statistical result of SA‐β gal positive cell number in Con, UVA, CGA and CGA‐probe group (scale bar = 50 μm, *n* = 3 samples per group), ***p < 0.001, **p < 0.01. (e, f) The mRNA relative expression of p16 (e) and p21 (f) in Con, UVA, UVA + CGA and UVA + CGA + P group (*n* = 6 samples per group), ***p < 0.001, *p < 0.05, ns:No statistical significance. (g) The labeling of situ protein labeling with CGA‐P in HDF cell. (h) Screening potential targets of anti‐photoaging of CGA. (i) Protein targets identified via ABPP in senescent HDF cells.

To identify the protein targets of CGA in HDF immortalized cell, the ABPP technology was used as previously reported (Zhu et al., 2022, 2024). We followed the flow chart as shown in Figure 2a to perform target identification of CGA. Firstly, a CGA chemical probe (CGA‐P) was designed and synthesized (Figure 2b). The alkynyl group was introduced to ‐COOH of CGA, enabling it to be linked to fluorescent dyes or biotin for labeling its binding protein. To assess whether the CGA probe exhibited similar biological activity to the original drug, we detect its ability to inhibit UVA‐induced cellular senescence. The results indicated that CGA‐P effectively reduced SA‐β‐gal staining and decreased the mRNA expression of p16 and p21 as CGA (Figure 2c–f), suggesting that this probe is suitable for subsequent target identification. Then, different concentrations of CGA‐P were incubated with UVA‐treated HDF cells, and cell lysates were reacted with a fluorescent dye through click chemistry to visualize the native targets of CGA. We found that the fluorescence intensity was enhanced with increased concentrations of CGA‐P (Figure 2g). Furthermore, co‐incubation with excess CGA competitively inhibited the 50 μM CGA‐P‐mediated fluorescence labeling signal in situ (Figure 2h). After sample preparation, Tandem Mass Tag (TMT) labeling and quantification by LC–MS/MS, high‐confidence proteins were selected based on criteria: (1) CGA‐P/DMSO enrichment ratio >4; (2) CGA‐P/CGA‐P+ CGA enrichment ratio >5; (3) statistical significance (*p* < 0.05). Among these proteins, Enolase 1 (ENO1) exhibited the greatest change in abundance compared to the competition group, thus warranting further investigation (Figure 2i). Collectively, ABPP technology combined with LC–MS/MS method was employed to identify ENO1 protein as a high‐confidence target of CGA in HDF cells.

### 2.3 CGA covalently binds to ENO1 by Cys399 residue

**FIGURE 3:**
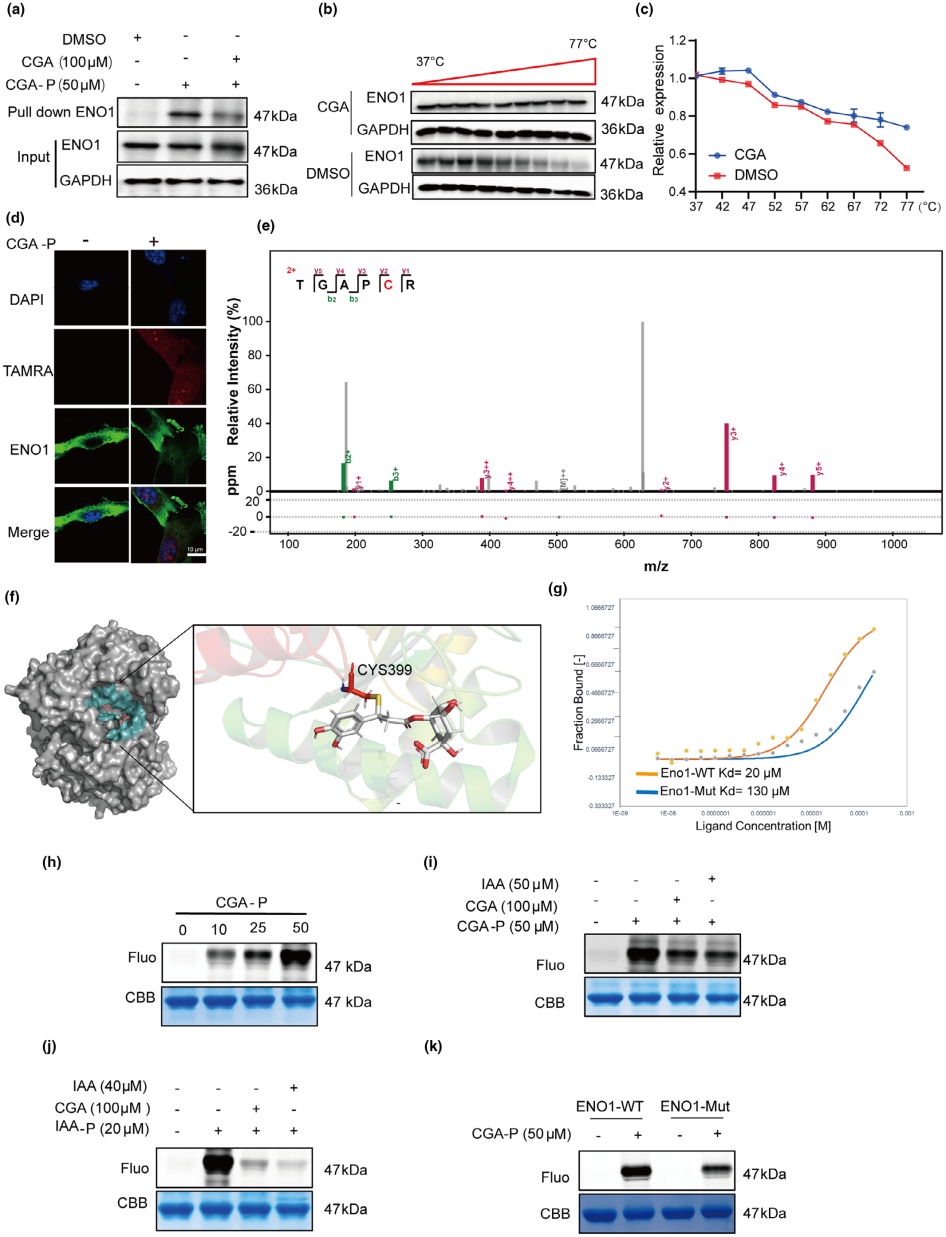
CGA can bind to the cysteine residue of ENO1. (a) CGA‐P pulldown ENO1 proteins, followed by Western‐blot. (b, c) The interaction between CGA and ENO1 proteins in lysis buffer of HDF cells confirmed by CETSA‐WB (b) and the statical result (c). (d) Co‐location CGA‐P and ENO1 proteins in HDF cells verified by immunofluorescence staining, (scale bar = 50 μm). (e) A profiling of CGA in activated HDF cells target Cys399 of ENO1 proteins. (f) A model of CGA binding with ENO1 proteins generated by molecular docking. (g) MicroScale Thermophoresis showing the binding kinetics between CGA and ENO1‐WT or ENO1‐C399A mutant. (h) CGA‐P labeling of rhENO1 proteins in a dose‐dependent manner. (i) CGA‐P labeling of rhENO1 proteins in the presence or absence of different competitors. (j) IAA‐yne labeling of rhENO1 proteins in the presence or absence of different competitor. (k) CGA‐P labeling of ENO1‐WT or ENO1‐C399A mutant in the presence or absence of CGA.

We conducted a series of experiments to verify the interaction between ENO1 and CGA. First, we performed a pull‐down assay and found that CGA can compete with CGA‐P for binding to the ENO1 protein in cells using its specific antibody by western blot (Figure 3a). Second, the cell heat transfer analysis (CETSA) was used to test whether the thermal stability of ENO1was affected by CGA. The results indicated that CGA significantly improved the thermal stability of ENO1 proteins in HDF cell lysate (Figure 3b,c). Third, we observed the co‐localization of ENO1 and CGA‐P‐mediated fluorescence mainly in the cytoplasm or membrane of HDF cells by immunofluorescence staining, which further validated their interaction (Figure 3d). We then explored the direct binding site of CGA on ENO1 protein by LC–MS/MS, and the results indicated that the Cys399 residue of ENO1 was covalently modified by CGA (Figure 3e). Molecular docking analysis also revealed that CGA binding sites include the Cys399 residue site of ENO1 (Figure 3f). To verify the above results, we carried out site‐directed mutagenesis (mutated Cys399 of ENO1 to Ala399) and purified both the wild type and mutant proteins. The MicroScale Thermophoresis (MST) results indicated that CGA exhibited a much stronger affinity with wild type ENO1 (Kd = 20 μM) than with mutant proteins (Kd = 130 μM) (Figure 3g).

Next, we performed an in‐gel fluorescence assay using the CGA‐P and recombinant proteins. We found that the ENO1 protein was labeled by CGA‐P in a concentration dependent manner (Figure 3h). Then recombinant proteins were pretreated with CGA or iodoacetamide (IAA; a compound reported to bind with cysteines) before incubating with CGA‐P or IAA‐P (IAA probe, a alkynyl group added to IAA). Our results showed that similar to CGA, IAA competed away CGA‐P or IAA‐P binding to proteins, suggesting that CGA was able to bind the cysteine residues of ENO1 (Figure 3i,j). Finally, we found that the wild‐type was significantly labeled by CGA‐P while the fluorescence intensity of mutant ENO1 proteins was largely impaired (Figure 3k). In summary, these data demonstrated that CGA covalently bound to ENO1 protein through Cys399 residue.

### 2.4 CGA inhibits cellular senescence by suppressing ENO1 and glycolysis pathway

**FIGURE 4:**
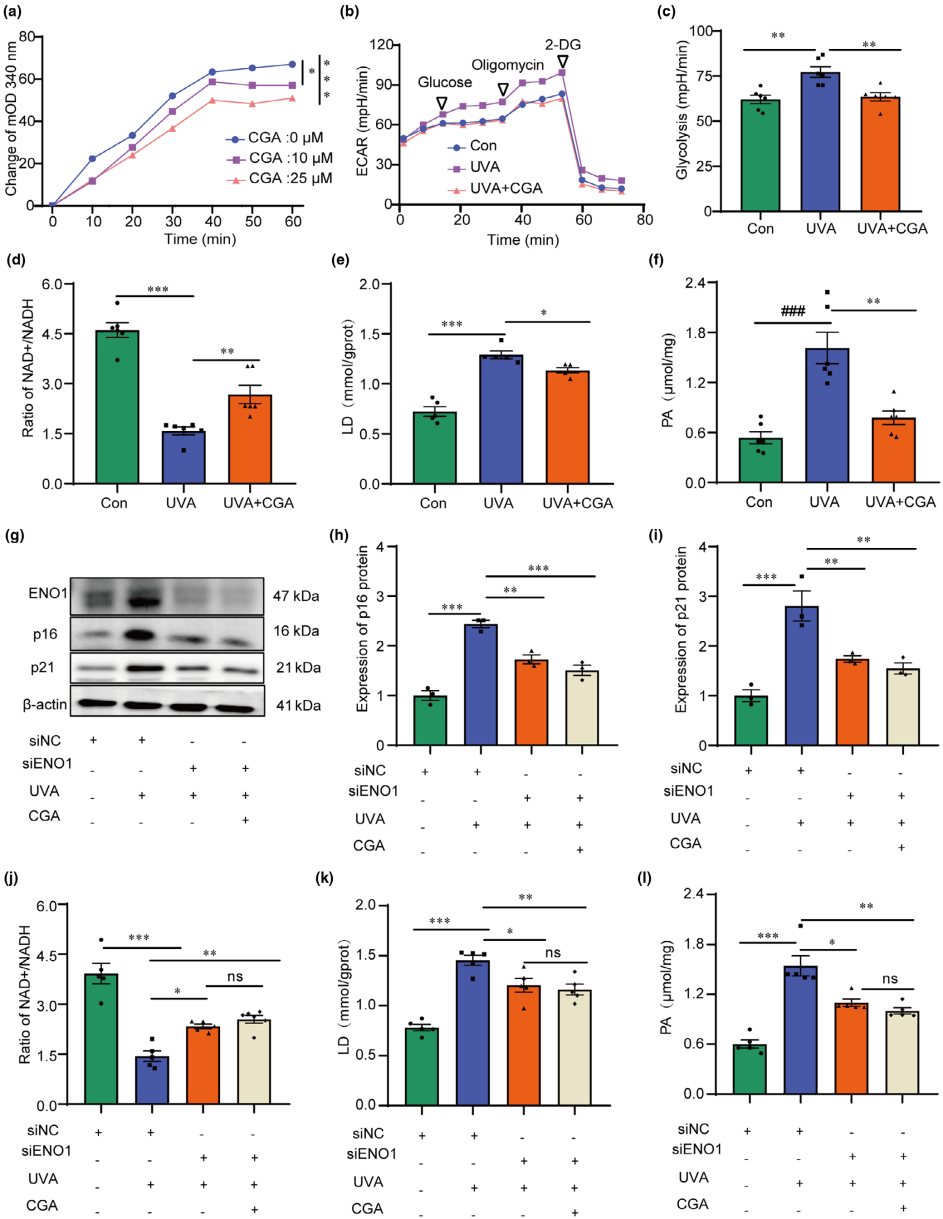
CGA targets ENO1 protein to inhibit glycolysis and thus inhibit photoaging. (a) CGA inhibits the enzymatic activity of ENO1 proteins (*n* = 7 samples per group), **p < 0.01, *p < 0.05. (b, c) The extracellular acidification rate (ECAR) (b) and glycolysis capacity of UVA‐induced HDF cells treated with CGA (c), (*n* = 6 samples per group), **p < 0.01. (d) The ratio of NAD+/NADH of UVA‐induced HDF cells treated with CGA (*n* = 6 samples per group), ***p < 0.001, *p< 0.05. (e) The level of lactic acid (LD) of UVA‐induced HDF cells treated with CGA (*n* = 5 samples per group), ***p< 0.001, *p< 0.05. (f) The level of pyruvic acid (PA) of UVA‐induced HDF cells treated with CGA (*n* = 6 samples per group), ***p < 0.001, **p < 0.01. (g–i) HDF cells transfected with siRNAs for ENO1 (si‐ENO1) with or without UVA, the level expression of ENO1, p16 and p21 protein under UVA or CGA conditions by western‐blot (*n* = 3 samples per group), ***p < 0.001, **p < 0.01, *p < 0.05. (j–l) HDF cells transfected with siRNAs for ENO1 (si‐ENO1) with or without UVA the level expression of NAD+/NADH, LD and PA under UVA or CGA conditions, (*n* = 5–6 samples per group), ***p < 0.001, **p < 0.01, *p < 0.05.

Since ENO1 is a glycolytic enzyme (Huang et al., 2022), we tested whether the binding of CGA to the protein affects the enzymatic activity. We found that CGA inhibited the activity of ENO1 proteins in a concentration‐dependent manner (Figure 4a). Additionally, extracellular acidification rate (ECAR) measurements revealed that UVA exposure increased glycolytic flux, while treatment with CGA significantly reduced ECAR and glycolysis (Figure 4b,c). Moreover, the NAD+/NADH ratio, lactate dehydrogenase (LD) levels and pyruvic acid (PA) levels were measured to evaluate the impact of CGA on glycolytic activity. The results indicated that CGA effectively inhibited glycolysis by increasing the NAD+/NADH ratio and decreasing LD and PA levels in UVA‐induced HDF cells (Figure 4d–f). To further elucidate the role of ENO1 in CGA‐mediated glycolysis suppression and cellular senescence inhibition, small interfering RNA (siRNA) was transfected into HDF cells to knock down ENO1 expression. We observed that silencing of ENO1 in UVA‐exposed HDF cells resulted in decreased expression levels of p16 and p21 proteins as well as reduced production of glycolysis pathway compared to the UVA‐induced group (Figure 4g–l). These findings suggested that CGA inhibits ENO1 activity, leading to suppression of glycolysis and senescence in UVA‐activated HDF cells.

### 2.5 CGA prevents UVR‐ induced skin photoaging in mice

**FIGURE 5:**
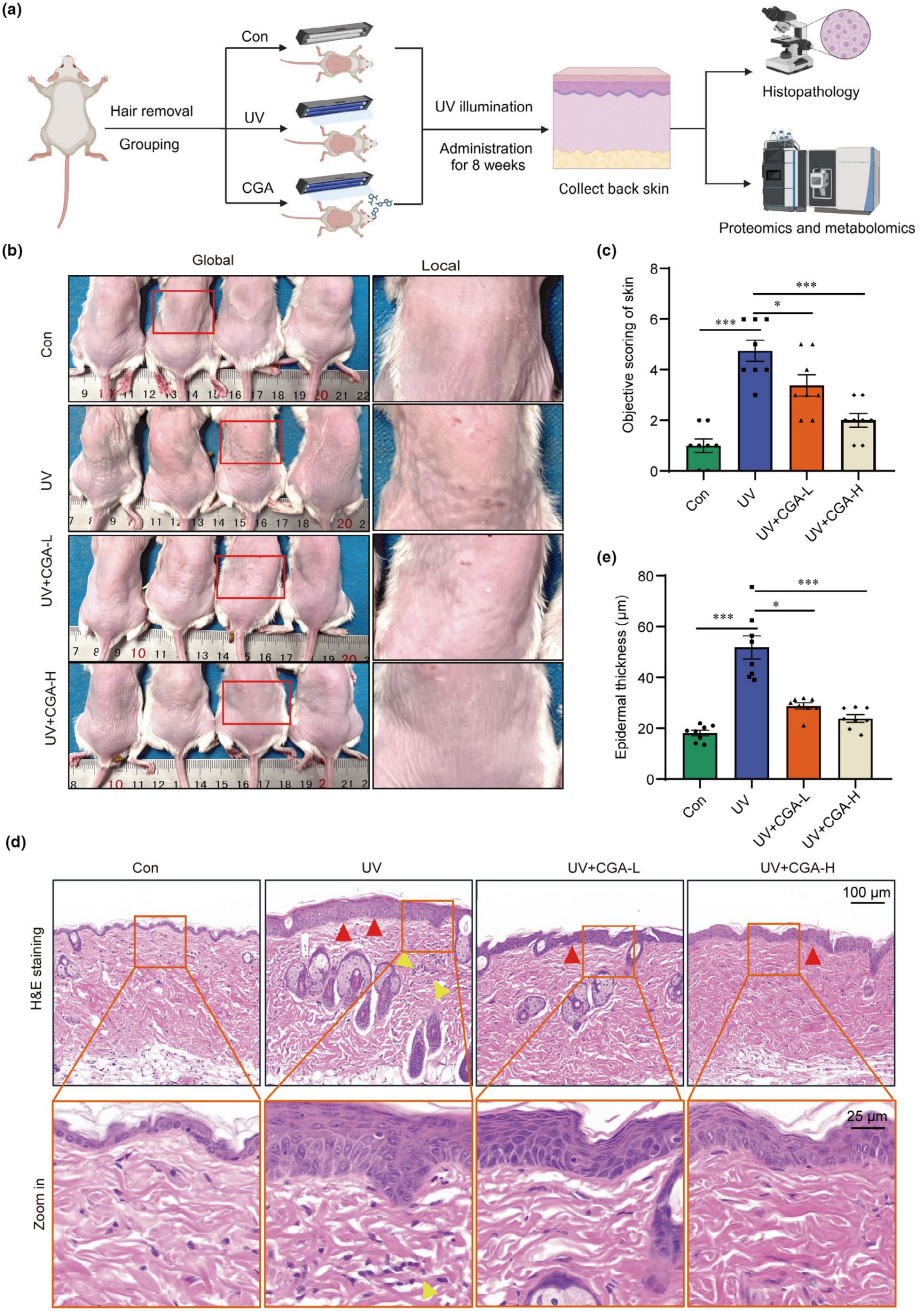
Chlorogenic acid improves UVR‐ induced skin photoaging in mice. (a) Schematic diagram of anti‐photoaging study of CGA in vivo. (b) Representative graph of wrinkle formation on the dorsal skin of mice at 8 weeks after indicated UVR treatment and CGA‐treatment. (c) Skin wrinkle score of dorsal skin of mice at Week 8 after indicated UV treatment and CGA‐treatment. (*n* = 8 samples per group), ***p < 0.001, *p < 0.05. (d) Representative graph of Hematoxylin–eosin staining of histological sections among Con, UV, UV + CGA‐L and UV + CGA‐H group, Red arrows indicate thickening of the epidermis and yellow arrows indicate inflammatory infiltration. (e) Statistics of epidermal thickness among Con, UV + UVA, UV + CGA‐L and UV + CGA‐H group (*n* = 8 samples per group), ***p < 0.001, *p < 0.05.

To confirm the anti‐photoaging effect of CGA in vivo, we established UVR‐induced photoaging mouse model as in a previous study (Khan et al., 2020). The model mice were treated with 25 and 100 mg/kg of CGA for 8 weeks as shown in Figure 5a. Wrinkles and reduced elasticity are both typical phenomena of skin photoaging, as the result of progressive dermal atrophy (Kong et al., 2013). Mice in control group had smooth dorsal skin with a few wrinkles, the back skin of mice in the UV group had severe wrinkles and some skin damage, while these phenotypes could be reduced after CGA treatment (Figure 5b). Specially, CGA could effectively reduce the skin wrinkle score in a dose‐dependent manner (Figure 5c). H&E staining results further indicated that compared to the control group, the UV group showed typical characteristics of skin photoaging, such as excessive keratinization of the stratum corneum, irregular thickening of the epidermis, and inflammatory infiltration of the dermis. Importantly, CGA treatment alleviates the skin pathological characteristics caused by UVR treatment (Figure 5d,e).

**FIGURE 6:**
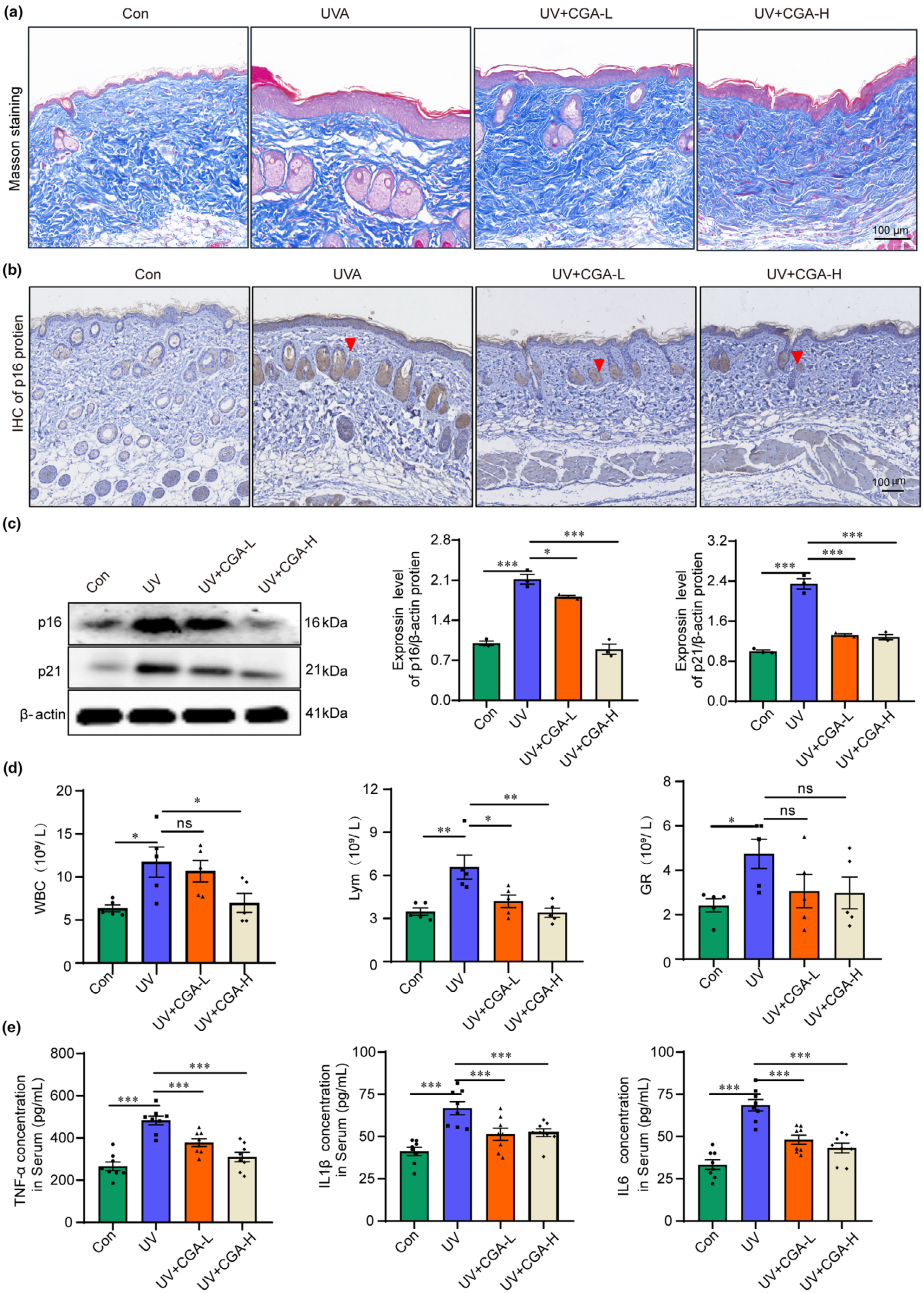
Chlorogenic acid inhibits the expression of UV‐induced aging marker and inflammation in mice. (a) Masson staining reflects the changes of skin collagen in mice skin among Con, UV, UV + CGA‐L and UV + CGA‐H group, scale bar = 100 μm. (b) Immunohistochemistry diagram of p16 proteins among Con, UV, UV + CGA‐L and UV + CGA‐H group, red arrows indicate positive expressions of p16 protein, scale bar = 100 μm. (c) The level expression of p16 and p21 proteins of dorsal back skin under UVR or CGA conditions by Western‐blot and statical results (*n* = 3 samples per group), ***p< 0.001, *p< 0.05. (d) Methods Blood routine shows the quantity of white blood cell (WBC), lymphocyte (Lym) and granulocyte (GR) under UV or CGA conditions, (*n* = 6 samples per group), **p < 0.01, *p < 0.05, ns: No statistical significance. (e) The concentration of inflammatory factor including TNF‐α, IL‐1β, IL‐6 in mice serum among Con, UV, UV + CGA‐L and UV + CGA‐H group, (*n* = 8 samples per group), ***p< 0.001.

Next, the photoaging and inflammatory state of the skin samples were evaluated. Masson staining was applied to show changes in skin collagen, and results showed that UV radiation resulted in decreased dermal collagen fiber content, irregular arrangement, and uneven distribution, which could be improved by CGA treatment (Figure 6a). IHC results showed that compared to the control group, mouse skin in the UV group had much higher expression of p16 proteins, which was effectively decreased by CGA treatment (Figure 6b). These results were further confirmed by detecting the protein level of p16 and p21 in skin samples by western blot analysis (Figure 6c). Besides, we found that CGA impeded the increasing amount of white blood cell (WBC), lymphocyte (Lym) and granulocyte (GR) induced by UVR, suggesting that the abnormal inflammatory state during photoaging was inhibited by CGA (Figure 6d). Moreover, the levels of TNF‐α, IL1β and IL6 in serum were notably decreased by CGA treatment (Figure 6e). These results demonstrated that CGA could improve UVR‐induced skin photoaging in mice.

### 2.6 CGA inhibits senescence in photoaging mouse model by proteomic analysis

To explore the global effect of CGA on protein expression in the photoaging mouse model, we performed a proteomic analysis, as shown in Figure S2a. Point diagram results showed that up‐ and down‐regulated proteins from UVA versus CON and UVA versus CGA in vivo and in vitro (Figure S2b). Then, a Venn diagram described the number of common and specific proteins of UVA vs. CON and UVA vs. CGA in vivo and in vitro (Figure S2c). Subsequently, GO enrichment analysis was performed based on all CGA‐regulated proteins. The results revealed that the up‐regulated pathways by UVA were associated with aging, including cell chemotaxis, oxidative stress and ROS, and we found CGA treatment inhibited the activation of these pathways (Figure S2d). The main histologic features of photoaged skin are a progressive decrease in collagen synthesis in aging dermal fibroblasts and changes in the composition of the extracellular matrix (ECM), with an increase in collagen degradation, which ultimately leads to a decrease in collagen content (Fitsiou et al., 2021). We found that the down‐regulated pathways by UV were involved in skin development and collagen activated signaling pathway, while CGA treatment could promote collagen‐activated pathways (Figure S2e).

Further, we delved into the potential mechanisms of CGA's resistance to photoaging in vivo. Previous studies have shown that UV induces high concentrations of ROS in the skin (Sreedhar et al., 2020). The gene set variation analysis (GSVA) analysis indicated that compared to the control and CGA treated groups, the Runing Enrichment Score of ROS metabolic process in the UVA group was increased (Figure S3a). Furthermore, we evaluated the enrichment score of four aging relative pathways: TNFA_SIGNALING_VIA_NKFB, INFLAMMATORY_RESONSE, TGF_BETA_SIGNALING and UV_RESPONSE_UP in three groups based on GSVA. Compared to the control group, the UV group had higher average score of four pathways, while CGA could reduce the average score induced by UVR (Figure S3a). We further downloaded a gene set of SASP to assess the ability of CGA to regulate SASP (Saul et al., 2022). The Heatmap showed that the protein levels of SASP factors were induced by UVR, while CGA treatment effectively inhibited their expression (Figure S3c). Notably, we further found that after CGA treatment, UVR‐upregulated glycolytic related proteins were down‐regulated (Figure S3d). In conclusion, proteomic analysis indicated that CGA inhibits senescence in the photoaging mouse model.

## 3 DISCUSSION

In the current study, we attempted to screen potential anti‐photoaging compound among natural products with the reported antioxidant and anti‐inflammatory activities based on our previous study (Zhang et al., 2022). We have demonstrated that CGA, as a natural polyphenol, plays an important role in inhibiting the photoaging process. On the one hand, CGA could effectively inhibit UVA‐induced HDF senescence, including reducing the proportion of SA‐β‐gal staining positive cells, the production of ROS, the expression of p16 and p21 proteins and the release of inflammatory factors. On the other hand, we evaluated the anti‐skin photoaging effect of CGA in vivo. We found that CGA not only alleviated the histopathological changes of UV‐induced skin aging in mice, but also reduced serum inflammation levels. These results demonstrate the potential of CGA as an effective compound against skin aging at both cellular and animal levels.

We pointed out that CGA can inhibit ENO1, a key enzyme of glycolysis, thereby resisting UVA‐induced skin photoaging. Based on the “click chemistry” theory (Parker & Pratt, 2020), we designed the probe of CGA and showed that CGA could bind to ENO1 in UVA‐induced HDF cell. We then provided a series of evidence to verify the interaction between CGA and ENO1 using a pull‐down assay, CETSA and immunofluorescence. Notably, CGA effectively inhibited the activities of ENO1 in a dose‐dependent manner, resulting in the decreased levels of NAD+/NADH, LD and PA which were reported as the products of glycolytic pathway (Chandel, 2021). Most importantly, studies have reported that the lactate level of senescent cells increases and the NAD+/NADH ratio decreases, which is consistent with our study. (Wiley et al., 2016). Most importantly, silencing ENO1 proteins expression in UVA‐stimulated HDF cells decreased p16 and p21 protein levels, further confirming that glycolysis is closely related to cellular senescence, which could be inhibited by CGA‐mediated ENO1 suppression. Although CGA has been previously reported to inhibit skin photoaging (Xue et al., 2022), our study for the first time clearly reveals its protein target and mechanism of action in senescent cells (Figure S4).

In recent years, cellular metabolic reprogramming has attracted significant attention in research areas such as cancer, inflammation and metabolic diseases (Abdel‐Wahab et al., 2019; Dimitropoulos et al., 2020; Li et al., 2023). And during the aging process, changes occur in the internal cellular environment, leading to alterations in energy metabolism patterns and increased reliance on the glycolytic pathway (Dou et al., 2023). It has been demonstrated that senescent cells display pyruvate dehydrogenase kinase 4 (PDK4)‐dependent increase in aerobic glycolysis and lactate production, which promote the growth of cancerous cell. Conversely, the inhibition of PDK4 has been shown to result in tumor regression, reduction in the severity of DNA damage and suppression of senescence‐associated secretory phenotype (SASP). Furthermore, it has been observed that senescent melanocytes demonstrate a notable elevation in glycolytic metabolic pathways, which may be linked to impaired mitochondrial function (Park et al., 2023). This indicates that metabolic reprogramming plays a pivotal role in melanocyte senescence, and that inhibiting the glycolytic pathway may prove an effective strategy for controlling this process. Although there have not yet been many studies exploring the direct relationship between photoaging and cellular metabolic reprogramming, it could be concluded that inhibition of key enzymes involved in the glycolytic pathway benefits the reduction the accumulation of excess metabolites, thereby attenuating cellular senescence and photoaging. Therefore, targeting the glycolytic pathway is probably a novel strategy to modulate cellular metabolism and slow down the development of photoaging.

In this study, we explored the pharmacological activity and protein targets of CGA in UV‐induced cellular models and a skin photoaging mouse model. We found that CGA prevented UVA‐induced cellular senescence by suppressing the activity of ENO1 protein to block the glycolytic pathway in vitro. To strengthen the potential of CGA as an aging therapeutic agent in clinical, further studies are required to confirm the mechanism of action of CGA via targeting ENO1 protein in vivo. Moreover, CGA may exert its anti‐aging effects by interacting with multiple targets identified in ABPP technology. It is important to note that future studies should continue to explore the mechanism of action of CGA and optimize the concentration and duration of its administration to bring this compound closer to the clinic.

## 4 MATERIALS AND METHODS

### 4.1 Drug

Chlorogenic acid was purchased from Aladdin (China Shanghai, 327‐97‐9).

### 4.2 Cell culture

SV40 large T‐antigen immortalized human skin dermal fibroblasts (HDF) were obtained from the Chinese National Cell Bank (Beijing, China). These cells were cultured in Dulbecco's Modified Eagle Medium (DMEM) with the addition of 10% Fetal Bovine Serum (FBS) and 0.1% penicillin–streptomycin and grown in a 37°C, 5% CO_2_ cell incubator. UVA was used to induce senescence of HDF cells, as reported in the literature with some modifications (Liu et al., 2022; Mu et al., 2022). Specifically, HDF cells were exposed to 10 J/cm^2^ UVA (UVP Crosslinker CL‐1000 L, Analytikjena) for 30 min, followed by treatment with candidate drugs, including CGA, CGA‐P, RA and so on for 24 h. Cells were collected for subsequent experimental studies.

### 4.3 SA‐β‐gal staining

HDF cells were treated with UVA stimulation and drug candidates, and then SA‐β‐gal staining was performed (kit purchased from Beyotime Biotechnology, Cat. No. C0602). Specifically, cells were fixed with paraformaldehyde for 15 min at room temperature. Subsequently, cells were rinsed three times with PBS, added with SA‐β‐gal staining solution, and then incubated at 37°C in a CO_2_‐free incubator for 16–20 h. At the end of the incubation, the cells were washed with PBS and the staining was visualized under a microscope.

### 4.4 ELISA assay

After UVA stimulation and candidate drugs treatment, the cell supernatant was used to detect the concentration of inflammatory factors including tumor necrosis factor α (TNF‐α) and interleukin‐6 (IL‐6) with enzyme‐linked immunosorbent assay (ELISA) kits (Mlbio, China Shanghai, mIC50536‐1, IC50325‐1), according to the manufacturer's instructions.

### 4.5 Western blot analysis

Total proteins were extracted from HDF cells using pre‐cooled RIPA buffer supplemented with a protease inhibitor cocktail. Subsequently, proteins were separated on 8%–12% SDS‐PAGE gels and electro‐transferred to PVDF membranes. Then, protein bands were blocked with 5% bovine serum albumin (BSA), and the proteins were extracted with primary antibodies (p16, Proteintech, 10,883‐1‐AP; p21, Proteintech, 10,355‐1‐AP; TNF‐α, Proteintech, 60,291‐1‐Ig; IL‐6, HuaBio, R1412‐2; ENO1, Proteintech, 11,204‐1‐AP) incubated overnight at 4°C, and incubated with the corresponding secondary antibodies for 1 h at room temperature. Finally, protein bands were developed with enzyme‐linked chemiluminescence (ELC) reagents. Protein amounts were analyzed semi‐quantitatively using ImageJ software.

### 4.6 Real‐time fluorescent quantitative PCR (RT‐PCR)

Total RNA was extracted from the samples using Trizol reagents. The RNA was reverse transcribed into cDNA, and the cDNA was served as the template for qPCR amplification. A real‐time PCR detection system, equipped with a thermal cycler and an optical detection module, was utilized to measure the fluorescence signal generated during each amplification cycle as the fluorophore bound to the target sequence. The relative expression of genes was calculated as 2^−∆∆Ct^. All primers used in the PCR were synthesized by Shanghai Sangon Biological (Shanghai, China), and the sequence of primers was shown in Table 1.

**TABLE 1:** Primer sequence for RT‐PCR.

Primer	Sequence (5′–3′)
p16 Forward	GATCCAGGTGGGTAGAAGGTC
p16 Reverse	CCCCTGCAAACTTCGTCCT
p21 Forward	TGTCCGTCAGAACCCATGC
p21 Reverse	AAAGTCGAAGTTCCATCGCTC

### 4.7 ABPP based targets identification

In our study, in order to analyze and identify cellular target proteins of CGA in situ, we performed Pull‐down experiments followed by LC–MS/MS or Western blotting analyses. The HDF cells were incubated with CGA for 4 h firstly, and then incubated with the CGA‐P for 1 h. At the end of the incubation, the cells were collected and the total protein of the cells was extracted. The click reaction mix was added to the protein solution and reacted on a Shaker for 1 h at room temperature. The protein solution was then precipitated with acetone at −20°C overnight. The precipitated proteins were dissolved in 1.5% SDS in PBS and incubated overnight at 4°C with an appropriate amount of streptavidin beads. The beads were then rinsed sequentially with 1% SDS, 0.1% SDS, 6 M urea and PBS. Subsequently, proteins enriched on the beads were reduced by dithiothreitol (DTT) and alkylated by iodoacetamide (IAA). The proteins were digested into peptides by adding trypsin immediately to the beads for more than 16 h at 37°C. The beads were then digested using a C18 chromatography column. The digested peptides were collected and desalted using a C18 column. The peptides were labeled with tandem mass tag (TMT) reagent and the labeled samples were pooled together. The pooled samples were identified using an Orbitrap Fusion Lumos mass spectrometer (Thermo Scientific, USA). For Pull‐down‐Western blot experiments, the procedures were similar to the pull‐down assay described above. After washing beads, loading buffer was added and denatured at 95°C for 10 min. Proteins content was analyzed by Western blotting for detection.

### 4.8 Cellular imaging

HDF cells were incubated with CGA‐probe and then fixed using 4% paraformaldehyde buffer for 10 min. Subsequently, the cells were permeabilized using 0.2% Triton X‐100 PBS solution for 20 min. The click chemistry mixture was added to the cells and reacted at room temperature for 1 h. Following this, the cells were stained with a fluorescence antibody (1:100 dilution) for 2 h and a nuclear dye (1:1000 dilution) for 10 min. The images were observed and recorded by confocal fluorescence microscopy.

### 4.9 Cellular thermal shift assay

To further confirm the target proteins of CGA, we performed cellular thermal shift assay (CETSA), an assay used to assess intracellular drug‐target interactions (Gao et al., 2022). First, equal amounts of proteins from HDF cells were incubated in CGA or DMSO for 2 h at RT, immediately followed by heating the proteins at temperatures ranging from 37°C to 77°C. Subsequently, the samples were centrifuged at 20,000*g*, 4°C for 30 min, and the supernatant was added to the loading buffer and denatured at 95°C for 10 min before performing Western‐Blot experiments. Finally, the target protein expression was analyzed semi‐quantitatively by ImageJ software.

### 4.10 Expression and purification of recombinant ENO1 protein

Wild‐type human ENO1 gene (NP_001419.1) and cysteine‐mutated ENO1 gene (Cys399 to Ala399, pET28a‐ENO1‐C399A) were cloned into pET28a vector, and then the plasmid was transformed into the expression protein of E. coli strain BL21. Specifically, BL21 was cultured at 180 rpm and 37°C in LB medium supplemented with 50 mg/mL kanamycin. The OD600 of the LB medium amounted to 0.8, isopropyl β‐D‐1‐thiogalactopyranoside (IPTG) was added to the medium and induced at 4°C for 17 h. The bacterial precipitate was then collected by centrifugation at 12,000 rpm for 30 min at 4°C and the proteins were extracted by the addition of lysis buffer (200 mM NaCl, 20 mM Tris–HCl, 1 mM PMSF) and sonication. Proteins were collected in the supernatant by centrifugation for 30 min at 12,000 rpm and 4°C and then purified using a Ni‐beads column (QIAGEN, Valencia, USA). The proteins were then eluted from the Ni‐beads column with a gradient of imidazole solution and the protein samples were concentrated. The purity and integrity of the recombinant protein were visualized using Coomassie Brilliant Blue (CBB) staining.

### 4.11 Molecular docking

The crystal structure of the protein was retrieved from the Protein Data Bank (PDB) (ENO1: PDB ID 3B97). Then, Discovery Studio Client was utilized for the dehydration and hydrogenation of the protein structures. We performed molecular docking with AutoDock Vina, a widely used molecular docking program that effectively predicts ligand binding patterns to protein targets. After the molecular docking was completed, Pymol software was used to map and visualize the docking results. By using these tools and software, the binding patterns and interactions of the target protein (ENO1) with the ligand (chlorogenic acid) can be predicted.

### 4.12 MicroScale thermophoresis

The purified ENO1 protein was added with RED dye from His‐Tag Labeling Kit RED‐tris‐NTA 2nd Generation (NanoTemper, SKU: MO‐L018), shaken well, then placed on ice and incubated for 30 min. 16 gradient concentrations of CGA solution were prepared, and then 10 μL of rhENO1 protein was mixed with it. Mixed samples were absorbed by capillary tubes for MicroScale thermophoresis (MST) experiments. The affinity between ENO1 protein and CGA drug was obtained by analysis software.

### 4.13 Activity assay of recombinant human ENO1 proteins

ENO1 assay kit (Abcam, USA, ab117994) was used to detect the inhibition of ENO1 activity by CGA. rhENO1 proteins (30 mmol/L) were incubated with DMSO or CGA (0, 10, 25 μM) for 1 h at room temperature, and the enolase activity was determined via a coupled reaction to the consumption of NADH in an assay buffer, according to the manufacturer's instructions.

### 4.14 Seahorse assay

A glycolysis stress test kit (103020‐100, Agilent Technologies, USA) was performed to assess the cellular extracellular acidification rate (ECAR) of HDF cells. HDF cells were cultured in Seahorse XFe96 microtiter plates, and cells were pre‐stimulated with UVA prior to CGA treatment for 24 h. The extracellular acidification rate (ECAR) of the cells was measured in real time on a Seahorse XFe96 extracellular flux analyzer (Agilent Technologies, USA) under basal conditions and after sequential addition of specific compounds that affect glycolysis. These compounds typically include glucose, oligomycin (an ATP synthase inhibitor) and 2‐deoxy‐D‐glucose (2‐DG, a glycolysis inhibitor). The data obtained were processed using the Seahorse XFe Wave software to assess the glycolytic activity and metabolic changes in HDF cells under CGA treatment.

### 4.15 Biochemical assays

HDF cells were cultured in six‐well plates with or without UVA and CGA treatment, cell lysis was used to detect the level of NAD+/NADH, LD and PA according to the manufacturer's instructions. NAD+/NADH detection kit was purchase from Beyotime (China Shanghai, S0175). Lactic acid detection kit was purchase from Nanjing Jiancheng (China Nanjing, A019‐2‐1). Pyruvate (PA) Content Assay Kit was purchase from Solarbio (China Beijing, BC2205).

### 4.16 RNA interference and transfection

Sequences of small interfering RNAs (siRNAs) were synthesized by Jing Rui Baikang (Beijing, China), and siRNAs interference and transfection methods were performed according to the manufacturer's instructions. HDF cells were cultured in DMEM without FBS and PS for 12 h. The siRNA vectors or NC vectors, along with packaging plasmids, were transfected into HDF cells using Lipofectamine 2000 After 6 h of transfection, the medium was replaced with fresh complete culture medium for subsequent experimental studies. The efficiency of transfection was periodically monitored by observing the expression of ENO1 proteins through Western blot analysis.

### 4.17 Animal

BALb/c mice (male, 18–22 g, 8 weeks), were raised on an ad libitum diet with a dark/light cycle of 12 h. The temperature was controlled at 24 ± 2°C and the humidity at 55 ± 5%. Animal experiments were approved by the Care and Use of Laboratory Animals Center of Shenzhen People's Hospital (Animal ethical code: AUP‐220516‐WJG‐0347‐01).

### 4.18 Photoaging model establishment and CGA treatment

The mouse skin photoaging model was established based on a previous report (Khan et al., 2020), with some modifications. The back hair of the mice was first removed with electric shaving, and then completely removed with hair removal cream, and the back skin was washed with normal saline. During the experimental period, the mice shed hair twice a week, before each UV exposure and CGA‐treatment. UVA (315–400 nm; Peak wavelength: 365 nm) and UVB (280 ~ 315 nm; Peak wavelength: 311 nm) rays were irradiated using an ultraviolet lamp at a distance of 20 cm from the mouse's skin. The intensity of UV exposure varied from week to week, as shown in the Table 2 below. In the experiment, the mice were divided into four groups: Con group, UV group, UV + CGA‐L group and UV + CGA‐H group. Among them, mice in Con group did not receive ultraviolet irradiation but vehicle solution (double distilled water) intragastric; mice in UVA group received ultraviolet radiation and vehicle solution intragastric; mice in UV + CGA group received ultraviolet radiation and given CGA solution intragastric (UV + CGA‐L: 25 mg/kg and UV + CGA‐H: 100 mg/kg). The mice were exposed to UVR and CGA‐treatment every day.

**TABLE 2:** Experimental design of UV exposure in this study.

Week	UVB (mJ/s)	UVB (s)	UVA (mJ/s)	UVA (s)
1	60	10	600	90
2	90	15	900	105
3	120	20	1200	180
4	150	25	1500	225
5	180	30	1800	270
6	210	35	2100	315
7	240	40	2400	360
8	360	60	3600	540

### 4.19 Evaluation of the appearance of back wrinkles in mice

The mice were anesthetized and the skin on their backs was captured using a digital camera at end of the experiment. The visual assessment of light damage score, as detailed in Table S1 in supplemental instrument, was utilized to evaluate the skin wrinkles of each mouse. This assessment was performed by two independent observers who were blinded to the protocol‐based grouping, as previously described (Kong et al., 2013). The observers assessed the extent of skin wrinkling on a scale ranging from 0 to 6, with 0 indicating no visible wrinkles or damage, and 6 indicating profound wrinkling. This standardized scoring system allowed for consistent and reliable evaluation of the skin's appearance across all experimental groups.

### 4.20 Hematoxylin and eosin (H&E) staining and epidermal thickening analysis

The skin samples were then fixed in 4% paraformaldehyde for 24 h and embedded in paraffin and sectioned into thin slices. Then, sections were deparaffinized and rehydrated using standard procedures, and H&E staining was performed according to established protocols. Lastly, the thickness of the mouse epidermis was evaluated by randomly selecting the distances of six sections under the optical microscope.

### 4.21 Immunohistochemical staining of p16 proteins

Paraffin‐embedded skin samples were successively cut into 4 μm slices, dewaxed, subjected to antigen repair, incubated with 3% hydrogen peroxide for 10 min and sealed with 3% BSA for 30 min. Primary antibody (p16, 30,519‐1‐AP, Proteintech, China) was incubated overnight at 4°C and then incubated with the secondary antibody, horseradish peroxidase‐ (HRP) for 1 h. DAB kit was used for color development and hematoxylin solution was used for nucleation. After these steps, the slices were analyzed with a neutral resin and inverted microscope.

### 4.22 Proteomics and data analysis

RIPA solution was used to extract proteins from cells (among Con, UVA, and CGA group) or skin tissue (among Con, UVA, and UV + CGA‐H group). Then, 5 mM DTT and 10 mM IAA were added into the proteinsolution and incubated in a 37°C shaker for 30 min. The proteins were digested overnight using trypsin at 37°C. The peptide solution was desalted by a commercial C18 column and detected by mass spectrometry.

The proteomic datasets generated by LC–MS/MS are statistically analyzed and visualized in R software. Firstly, we filtered the proteins with low quality. In addition, the mean value was used to fill in the missing values based on the abundance values of the same set of samples. Finally, use limma for DEPs analysis, clusterprofiler for GO analysis or GSVA analysis.

### 4.23 Statistical analysis

Statistical analysis was performed using GraphPad Prism 8.0 software, and data are expressed as means ± SEM. The significance of differences was evaluated by Student's *t*‐test between groups or by one‐way ANOVA followed by the Tukey's test in multiple groups. A *p*‐value less than 0.05 (*p* < 0.05) was considered statistically significant.

## AUTHOR CONTRIBUTIONS

JGW and YHZ designed and supervised this work; XLH, CW and QYZ performed the experiment and analyzed the data; TY, QYG, and YXW provided statistical analysis; JYG, PJG, JZZ, and TH provided acquisition, analysis and interpretation of data, and statistical analysis; XLH and YHZ wrote the paper. All authors read and approved the final paper.

## FUNDING INFORMATION

The work was supported by grants from the Scientific and Technological Innovation Project of the China Academy of the Chinese Medical Sciences (CI2023E002‐Y‐30, CI2023E005TS01, CI2023D003, CI2021B014, CI2021A05101, and ZG2024001‐05), the National Key Research and Development Program of China (2020YFA0908000), the Innovation Team and Talents Cultivation Program of the National Administration of Traditional Chinese Medicine (ZYYCXTD‐C‐202002), the National Natural Science Foundation of China (32201177, 82141001, and 82074098), the Fundamental Research Funds for the Central Public Welfare Research Institutes (ZZ14‐YQ‐061, ZZ16‐ND‐10‐16; ZZ17‐ND‐10‐15, ZZ16‐ND‐10–24 and ZZ16‐ND‐10‐16) the Science and Technology Foundation of Shenzhen (Shenzhen Clinical Medical Research Center for Geriatric Diseases), and the Shenzhen Medical Research Fund (B2302051).

## CONFLICT OF INTEREST STATEMENT

Authors declare that they have no conflict of interest.

## Supporting information


Data S1.

## Data Availability

The data reported in this paper have been deposited in the OMIX, China National Center for Bioinformation / Beijing Institute of Genomics, Chinese Academy of Sciences (https://ngdc.cncb.ac.cn/omix: accession no. OMIX006532).
