# Supplementary material for: Identifying ENO1 as a protein target of chlorogenic acid to inhibit cellular senescence and prevent skin photoaging in mice
Source: Aging Cell. 2024 Dec 31;24(4):e14433. doi: 10.1111/acel.14433 (PMC11984691; doi:10.1111/acel.14433)
Supplement: Supplementary file 1 — Data S1. [file ACEL-24-e14433-s001.docx]

**Supplement Figure**


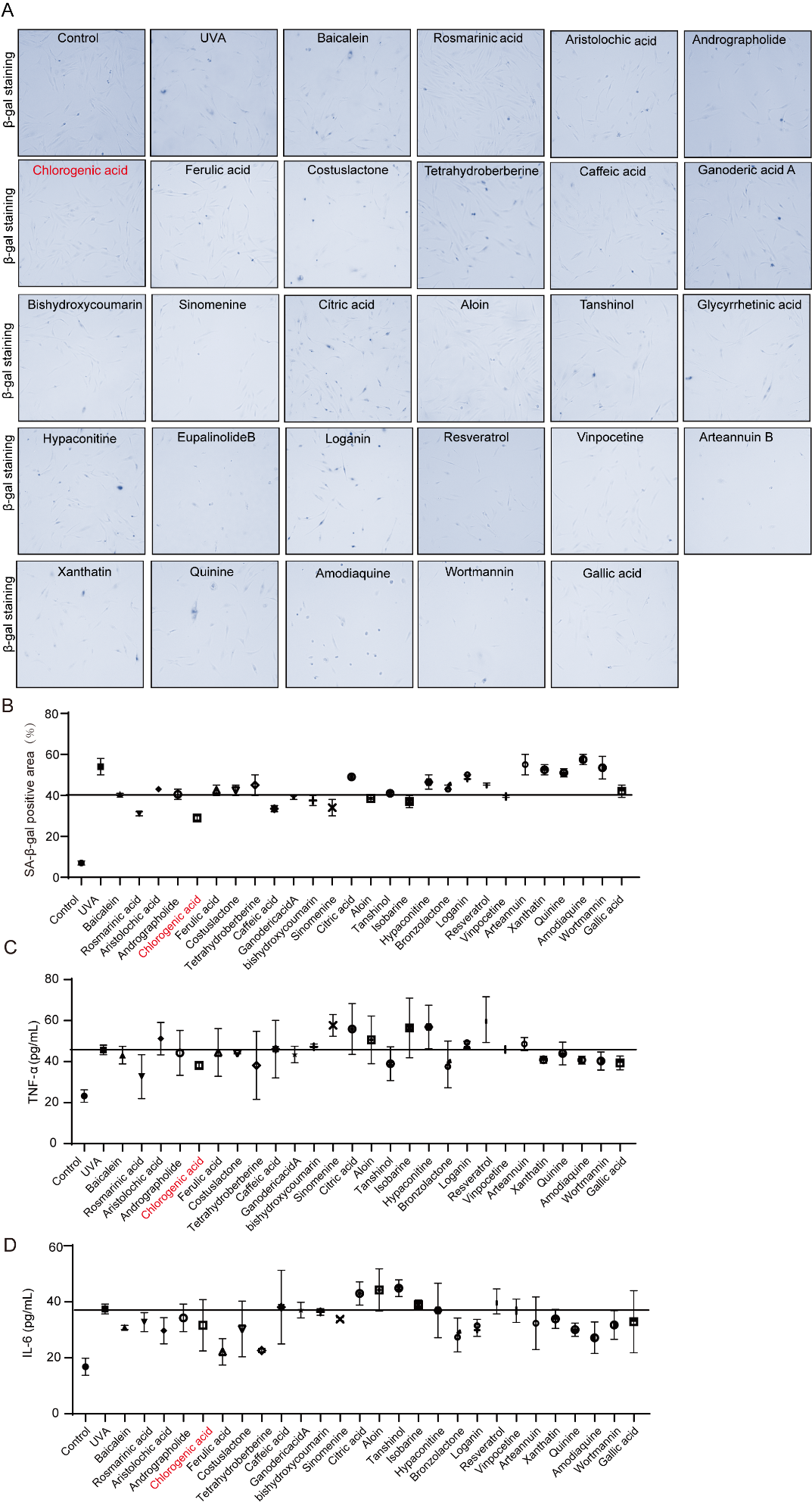


**Supplement Figure 1 Screening of active molecules to prevent UVA-induced senescence of HDF cells. A and B.** The representative graph of SA-β gal staining of UVA-induced HSF cell under different drug effects and statistics. **C.** The level of TNF-α of UVA-induced HSF cell supernatant under different drug effects**.** **D.** The level of IL-6 of UVA-induced HSF cell supernatant under different drug effects.


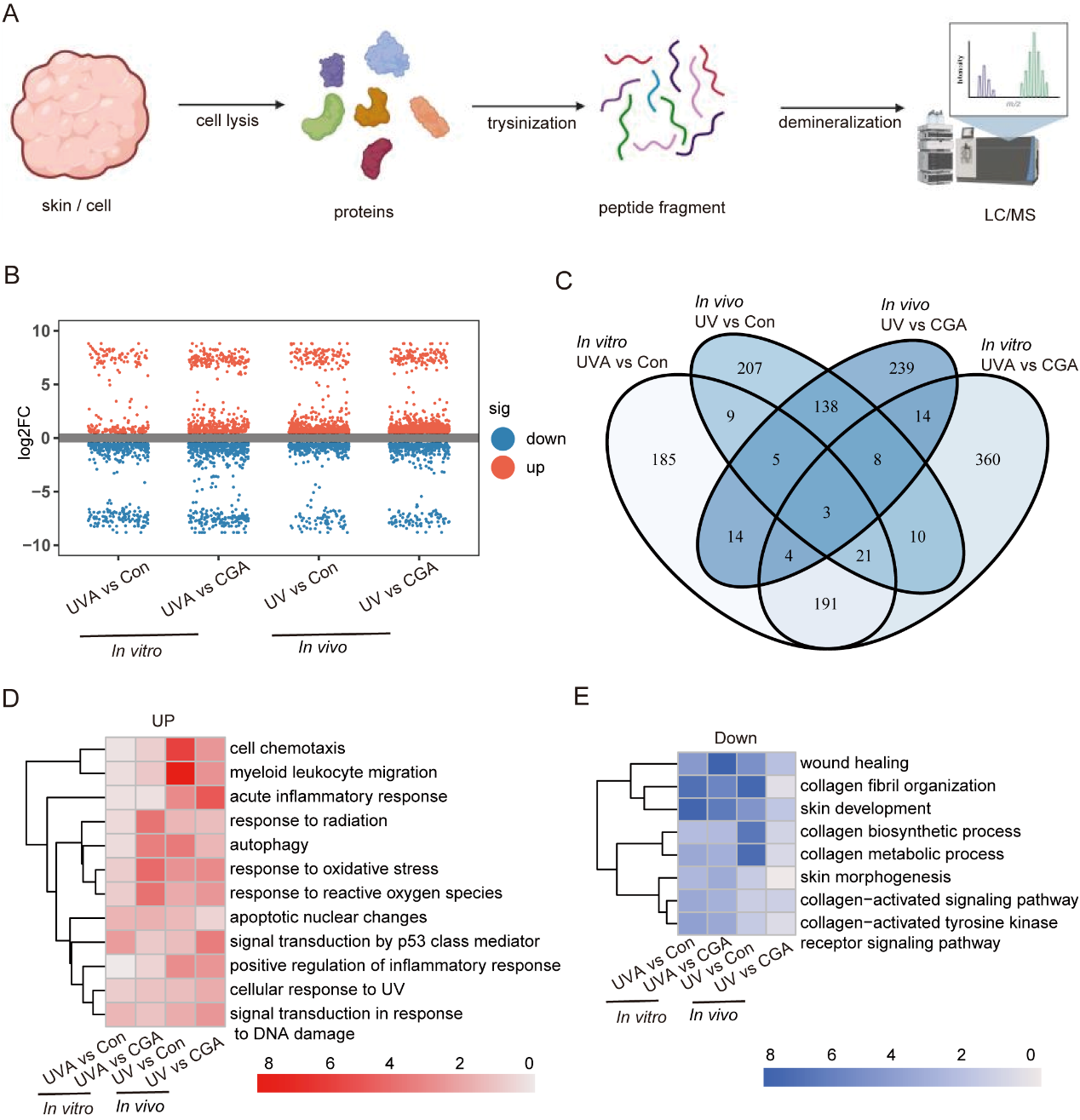


**Supplement Figure 2 Proteomic revels Chlorogenic acid inhibits photoaging *in vitro* and *in vivo* of UVR-induced model. A**. Schematic diagram of anti-photoaging study of CGA through proteomic. **B**. The plots show up-regulated proteins (red dots) and down-regulated proteins (blue dots) involved in CGA-treated *in vitro* and *in vivo* of photoaging model. **C**. Venn diagram shows the common and unique of proteins in CGA-treated *in vitro* and *in vivo* of photoaging model. **D**. The heatmap shows up-regulated pathway in CGA-treated *in vitro* and *in* *vivo* of photoaging model. **E**. The heatmap shows down-regulated pathway in CGA-treated *in vitro* and *in vivo* of photoaging model.


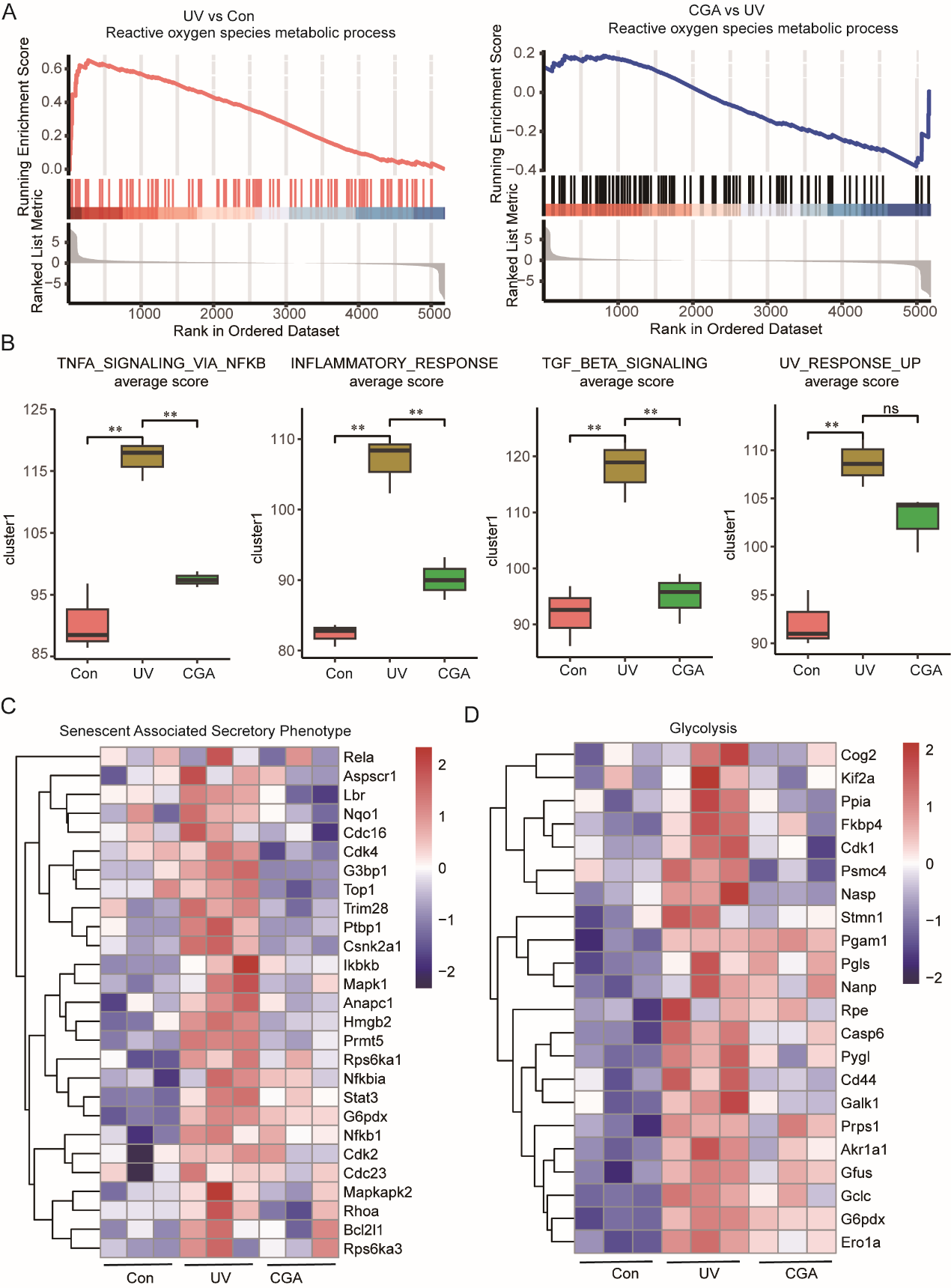


**Supplement Figure 3. Proteomic revels anti-photoaging of Chlorogenic acid relating inflammation and glycolysis *in vivo* model.** **A**. GSVA analysis shows the scores of reactive species metabolic process in UVA vs Con and CGA vs UVA *in vivo*. **B**. GSVA analysis shows the average score of TNFA_SIGNALING_VIA_NFKB, INFLAMMATORY_RESPONE, TGF_BETA_SIGNALING and UV_RESPONE_UP in each group. **C**. The heatmap of proteins of senescent associated secretory phenotype in among Con, UVA, and CGA-H group. **D**. The heatmap of proteins of Glycolysis among Con, UVA and CGA-H group.


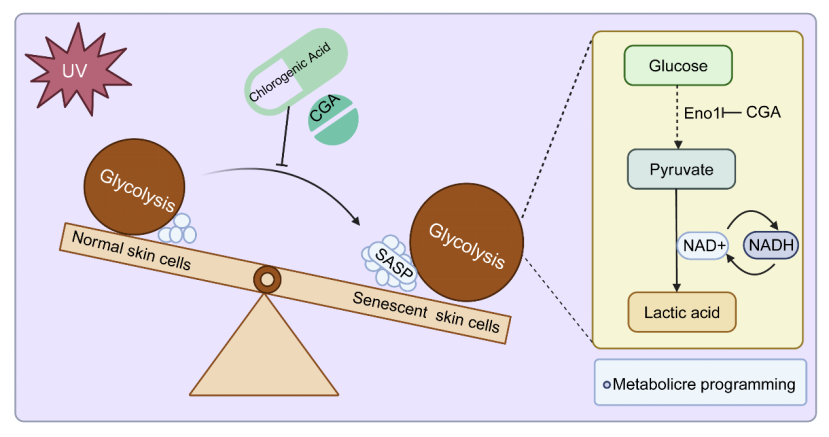


**Supplement Figure 4. Schematic model showing the molecular mechanisms of the UV-induced photoaging under chlorogenic acid-treatment.**

**Supplement Table**

Table S1. Score scale for evaluation of skin wrinkles

| **Score** | **Descriptions** | **Pictorial examples** |
| --- | --- | --- |
| 0 | Smooth skin | 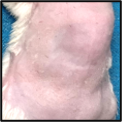 |
| 1 | Fine wrinkle | 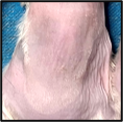 |
| 2 | A little bit shallow wrinkles | 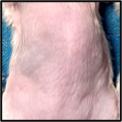 |
| 3 | Shallow wrinkles across the dorsal skin | 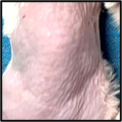 |
| 4 | Deep and coarse wrinkles with laxity | 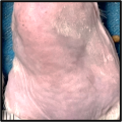 |
| 5 | Increased wrinkle dept | 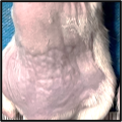 |
| 6 | Severe wrinkles with skin damage occurs | 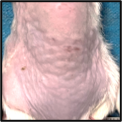 |
